# Implementing Interoperability in the Seafood Industry: Learning from Experiences in Other Sectors

**DOI:** 10.1111/1750-3841.13742

**Published:** 2017-08-21

**Authors:** Tejas Bhatt, Martin Gooch, Benjamin Dent, Gilbert Sylvia

**Affiliations:** ^1^ Inst. of Food Technologists 818 Connecticut Ave., Suite 850 Washington, DC 20006 U.S.A; ^2^ Value Chain Management Intl. Inc. 1155 North Service Rd. West, Suite 11 Oakville ON L6M 3E3 Canada; ^3^ Coastal Oregon Marine Experiment Station Oregon State Univ. Hatfield Marine Science Center, 2030 Marine Science Drive Newport OR 97365 U.S.A

**Keywords:** interoperability, seafood industry, supply chain, technology architecture, traceability

## Abstract

Interoperability of communication and information technologies within and between businesses operating along supply chains is being pursued and implemented in numerous industries worldwide to increase the efficiency and effectiveness of operations. The desire for greater interoperability is also driven by the need to reduce business risk through more informed management decisions. Interoperability is achieved by the development of a technology architecture that guides the design and implementation of communication systems existing within individual businesses and between businesses comprising the supply chain. Technology architectures are developed through a purposeful dialogue about why the architecture is required, the benefits and opportunities that the architecture offers the industry, and how the architecture will translate into practical results. An assessment of how the finance, travel, and health industries and a sector of the food industry—fresh produce—have implemented interoperability was conducted to identify lessons learned that can aid the development of interoperability in the seafood industry. The findings include identification of the need for strong, effective governance during the establishment and operation of an interoperability initiative to ensure the existence of common protocols and standards. The resulting insights were distilled into a series of principles for enabling syntactic and semantic interoperability in any industry, which we summarize in this article. Categorized as “structural,” “operational,” and “integrative,” the principles describe requirements and solutions that are pivotal to enabling businesses to create and capture value from full chain interoperability. The principles are also fundamental to allowing governments and advocacy groups to use traceability for public good.

## Introduction

The seafood industry is increasingly competitive, global, and complex. Consumers are demanding verifiable information on the source, quality, and safety of the products they chose to consume. Governments are placing increasingly stringent compliance requirements on businesses, regardless of where they are geographically located and their role in the supply chain.

These macrodrivers of change are forcing businesses in the seafood industry to manage an expanding array of increasingly onerous risks. The ability of businesses to proactively manage risks, reduce costs, and increase revenue depends on the effective sharing of information. Verifying the accuracy and rigor of data exchanged within and between businesses depends on the existence of effective interoperable information systems. Effective interoperability relies on the systems used by businesses operating along the supply chain to share a common technology architecture—in other words, a common blueprint or framework.

With traceability being a fundamental requirement for businesses to effectively manage their operations and business relationships, the Institute of Food Technologists’ (IFT) Global Food Traceability Centre (GFTC) is working to identify the most effective means of establishing a technology architecture suited to full chain interoperability in the seafood industry. The GFTC project stems from a growing realization that a need exists to establish a global, secure, interoperable seafood traceability system. Establishing effective global traceability systems relies on the development of a cohesive and consistent approach to the delivery of information technology capabilities and functions. An information technology architecture describes the process of achieving this through methodical development of a common and coherent series of specifications, protocols, guidelines, and concepts.

Technology architectures are developed by engaging industry stakeholders in purposeful dialogue about why an architecture is required, the benefits and opportunities that an architecture offers industry, and how the architecture will translate into practical results. This article describes lessons learned from the sectors who are ahead of the seafood industry in enabling interoperability, with the goal of fostering the dialogue, momentum, and activities needed in the seafood industry to develop and implement the technology architecture that is required for global interoperable seafood traceability.

We begin by describing the benefits that individual businesses, industries, and other stakeholders can achieve from interoperability. We then describe 4 industries—finance, travel, fresh produce, and health care—that have either implemented interoperability or which are in the process of enabling it. We include the processes being followed to achieve interoperable traceability and key lessons learned. While these industries vary analogously compared to seafood, each of the examples provides insights from which the seafood industry can benefit. This report then describes principles that we identified as critical to enabling effective, efficient interoperability, and which must be reflected in a technology architecture for full chain seafood traceability.

## Benefits of Interoperability

Although evidence gathered from GFTC projects (for example, Sterling and others 2015) and other research shows intuitively and anecdotally that the benefits of interoperability can be significant, most of the reported benefits that businesses and industries have achieved in implementing interoperable information systems are primarily qualitative.

The fact that few studies have been undertaken to quantify the benefits of interoperability, thereby limiting the development of a rigorous business case for establishing interoperability, is reflected in the following statements:

- “(A) comprehensive evaluation of the business impact of interoperability is still lacking.” (INSEAD 2006)
- “It is widely believed that the establishment of interoperability of the information systems of a firm with those of its collaborators (for example, customers, suppliers, and business partners) can generate significant business value. However, this has been empirically investigated only to a very limited extent.” (Loukis and Charalabidis 2013).

### Current limitations

Sterling and others (2015) along with other researchers (for example, EC 2008) have identified a number of reasons why few efforts have been made to quantify the benefits of implementing interoperability. These include:

- Work on interoperability and traceability has focused on addressing technical issues.
- The return on investment (ROI) from interoperability and traceability will vary significantly depending on the underlying characteristics of each business and supply chain, and the scope/objectives for investing in interoperability and traceability.
- ROI is generated from both operational impacts and strategic impacts. Operational impacts (for example, costs, revenue, market size/value, quality, and transaction costs) are typically easier to quantify than strategic impacts. Yet, it is the latter which are recognized as a main factor that triggers investment decisions, because they are a better indicator than ROI calculations of how a firm will generate long‐term benefits (Porter 1985; Prahalad and Hamel 1990).
- Trying to compare ROI is difficult because it would require assessing the degree of interoperability in each system being compared and the environment into which it was implemented. The ROI from traceability will be affected by the extent to which it is imbedded in a business’ or supply chain's operation and management systems, as well as the environment in which the system operates. These factors are highly variable and hard to measure.
- Interoperability itself, as for traceability, does not generate the ROI. Rather, it facilitates increased efficiency/effectiveness of existing business processes (such as traceability systems, inventory management, customer/consumer responsiveness, and innovation, including new product development). Thus, the ROI depends on the effective/efficiency of each business process prior to implementation, and to what extent performance can be improved (Ford and Ogden 2010).
- Given the forgoing factors, an industry level analysis would produce findings too generic for transferable conclusions; and findings from case studies of individual businesses and supply chains would be too specific and thus ungeneralizable. To be helpful, therefore, studies must balance industry‐ and enterprise‐level considerations. The lack of a solution to this problem negatively impacts the willingness of potential funders to support an initiative designed to quantify the benefits of interoperability and traceability.

Further, in terms of quantifying the benefits of interoperability, the purpose and concept of interoperability extends beyond traceability, potentially encompassing all manner of computerized and electronic information systems. Intuitively and anecdotally, the more that businesses operating along a supply chain implement interoperable systems, the greater the potential impact on operational and strategic performance. The more diffuse a system's impact, however, the more difficult it is to isolate the impact from other potential determining factors, and therefore the more challenging it is to measure benefits with any sense of accuracy.

### Reported benefits of interoperability

Attempts to identify the financial benefits of interoperability include 2 significant studies. Described briefly below, the first study examined the U.S. automotive industry. The second looked into the European manufacturing and service sectors. These and other, less expansive research (for example, case studies of the produce traceability initiative [PTI]), indicate that the benefits of interoperability primarily stem from 3 sources of competitive advantage, which are:

- New or improved products and services (for example, greater functionality or customer satisfaction)
- Innovative forms of business cooperation (for example, collaborative product design) and
- More effective supply chain management (for example, reduction of operating costs, increase in quality).

#### U.S. automotive industry

A study of the U.S. automotive industry conservatively estimated that imperfect interoperability costs the U.S. industry more than $1 billion/year and delays introduction of new vehicles by at least 2 mo (Brunnermeier and Martin 2002).

The potential savings identified by this research included $295 million on inventory and freight costs, and $198 million on the supply chain coordination process and interoperability tool maintenance.

#### Multiple European industries

The second study (Loukis and Charalabidis 2013), which was completed for the European Commission, examined the experiences of decision makers from more than 14,000 firms in the manufacturing and service sectors, including food and beverage, across Europe. The size of respondents’ operations ranged from micro‐businesses to multinational businesses.

Entitled “Advanced Technologies for Interoperability of Heterogeneous Enterprise Networks and their Application” (ATHENA), the European research examined the effect of adopting the 3 main types of interoperability standards (industry specific, proprietary, and Extensible Markup Language [XML]‐based) on 4 dimensions of business performance (financial, customers, internal business processes, and learning and innovation).

The ATHENA report concluded that industry‐specific interoperability standards had the greatest impact, because they had the appropriate depth and breadth for the particular industry. Industry‐specific standards also offered a high level of applicability, resulting in greater value generation.

## Finance Sector

The scale, geographic location, and capabilities of businesses operating in the financial industry differ enormously: from publicly‐owned, multinational banks with assets totaling trillions of dollars and employing hundreds of thousands of employees, to independent privately‐owned wealth management companies whose assets are minuscule in comparison. The size of the financial transactions conducted by these businesses range enormously in size. Enormous differences also exist in the geographic location and distances across which these transactions are sent or received the currencies exchanged and the degree to which transactions are aggregated or disaggregated as they move between individuals or institutions. The complexity of the financial sector has increased with the introduction of online banking, internet banks, and account‐2‐account (A2A) transactions, which require collaboration between commercial businesses who are often competitors (Clark and Camner 2014). An increasingly stringent regulatory environment—including legal compliance and oversight systems implemented by international, national, and regional authorities—has also increased the complexities and challenges faced by organizations operating in the financial sector.

IS0 20022 is a methodology that the financial industry uses to harmonize previously non‐interoperable formats and systems to establish “a collection of ‘message definitions’ and a process of how these can be applied to specific business domains.” ISO 20022 is a global and open standard that is not controlled by a single interest, which is available to anyone in the industry who wants to participate and free for anyone to implement on any network. The methodology has well‐established processes from a maintenance, evolution, and governance perspective. The adoption, implementation, and evolution of the ISO 20022 processes and procedures is overseen by the Society for Worldwide Interbank Financial Telecommunication (SWIFT).

### Governance

**Figure 1:**
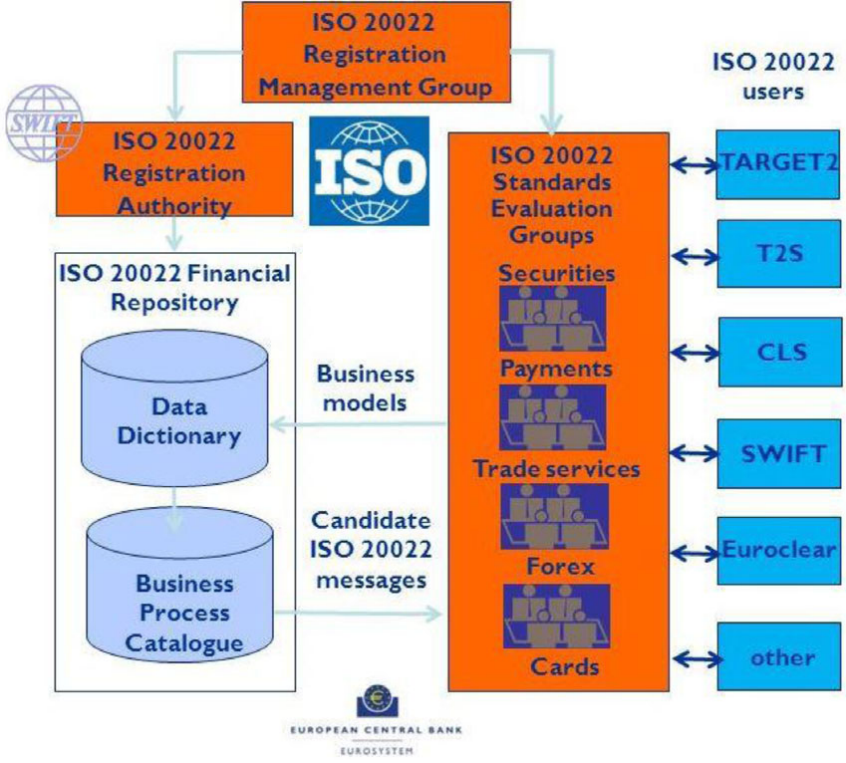
ISO 20022 governance (European Central Bank 2013; with permission)

The governance system established to manage the ISO 20022 process is shown in Figure 1. The governance system is comprised of 4 interdependent groups:

- Registration management group
- Registration authority (SWIFT)
- Standards evaluation groups
- Technical support group (not shown in diagram)

#### Registration management group

The ISO 20022 Registration Management Group (RMG) is made up of industry experts nominated by registered members. The RMG is the overarching body, supervising the overall registration process. The group reports to ISO Technical Committee 68, which oversees all financial services standards, and is responsible for ensuring that ISO 20022 is a trusted standard for systems for the exchange of information for financial services, and for promoting/supporting the involvement of financial service stakeholders to facilitate the registration and maintenance of these systems.

The role of the RMG is to:

- Define the scope of standards evaluation groups (SEGs)
- Approve business justifications for new messages, then  allocate them to one or more SEGs
- Act as a “court of appeal” for conflicts between the registration authority, the technical support group, the SEGs and organizations that want to develop ISO 20022 messages. Someone that wants to introduce a new financial message format or system is known as a “submitting organization.”

#### Registration authority

SWIFT is a member‐owned cooperative and, with more than 11000 clients and customers, it is the largest provider of secure financial messaging services to banking and securities organizations. SWIFT brings together the financial community at global, regional, and local levels to shape market practice, define standards, and debate issues of mutual interest or concern. SWIFT also offers products and services to facilitate access and integration, identification, analysis, and regulatory compliance. SWIFT does not transfer funds itself. The purpose of SWIFT is to ensure the standardization that results in the highly efficient, effective, and rigorous exchange of funds through electronic means (Benson and others 2014).

SWIFT's recommended framework, that organizations who wish to introduce new financial messages follow, is:

- Understand how different market infrastructure adoption approaches have an impact on the implementation options for financial institutions;
- Evaluate the relative merits of a tactical versus strategic implementation;
- Make informed decisions about the implementation roadmap, based on business and technology impact assessments, and create an enterprise architecture that is as ‘future‐proof’ as possible; and
- Understand how SWIFT can help throughout the process, from evaluation and design to implementation.

In addition to overseeing ISO 20022 related activities, SWIFT has a close working relationship with organizations established to provide regulatory oversight to the finance industry. These regulatory oversight bodies include the Financial Action Task Force, which sets and enforces global standards for combating financial crime, and the Basel Committee for Banking Supervision, which issues financial transaction compliance standards that countries must implement. This relationship ensures that interoperability solutions developed for the finance sector incorporate best practice systems for meeting current and anticipated regulatory requirements. The close relationship with regulatory oversight bodies also ensures that the design of interoperable solutions incorporates a mechanism that enables them to evolve as regulations and compliance requirements change and, conversely, compliance regulations and enforcement practices to reflect industry needs and changing technologies.

#### Standards evaluation groups

SEGs are made up of industry experts representative of future users, each with a remit to:

- Ensure that the right industry groups are informed of proposed developments, and all business requirements will be addressed.
- Validate the newly developed message definitions from a business perspective, ensuring that what will be posted in the ISO 20022 repository by the RA addresses the needs of future communities of users as described in the business justification accepted by the RMG.
- Approve changes to existing message definitions.

#### Technical support group

The technical support group (TSG) comprises experts in the technical implementation of the ISO 20022 standard. The TSG provides technical support to the other registration bodies (RMG, RA, and SEGs). The TSG also provides technical support to submitting organizations and users.

More specifically, the TSG:

- Advises submitting organizations, the RA, and SEGs on interpretation of the standard's methodology and compliance;
- Assists an SEG on technical issues arising from the evaluation of candidate ISO 20022 messages, or the technical “implementability” of the proposed messages; and
- Comments on any proposed adoption of new technical specifications, or the best way to organize migration to new technical specifications.

### Development of new specifications

**Figure 2:**
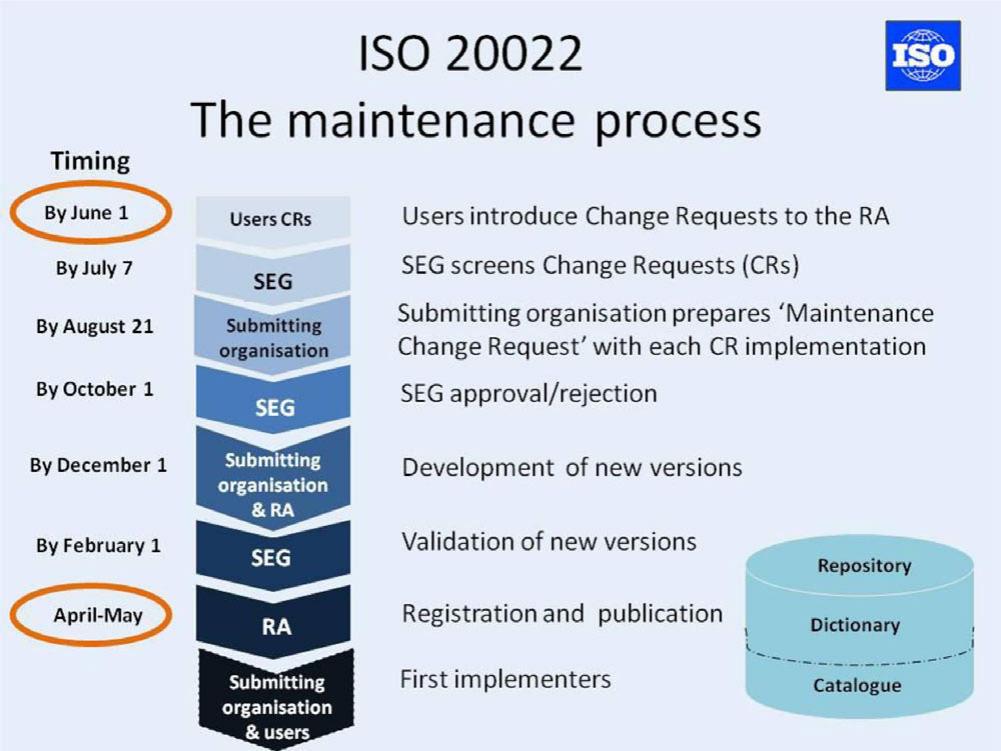
Implementation of changed messaging (ISO 2016; with permission).

The amount of time taken for a submitting organization's proposed change in messaging to be introduced into the market is typically 12 mo (ISO 2016). Figure 2 shows an example of a change‐schedule timetable and associated activities/deliverables.

Depending on the anticipated impact on industry's regular operation and whether a fundamental or incremental change is involved, the implementation of new messaging follows 1 of 3 routes:

- Big bang, with a single mandatory migration deadline;
- Phased, with multiple migration phases and deadlines, or
- “Free,” with individual suppliers deciding when to migrate.

### Interoperability implementation process

SWIFT's experience with major initiatives across multiple geographies to enable and encourage adoption of harmonized practices in an environment where no 2 businesses are alike led it to establish a best‐practice approach to implementing interoperability.

**Figure 3:**
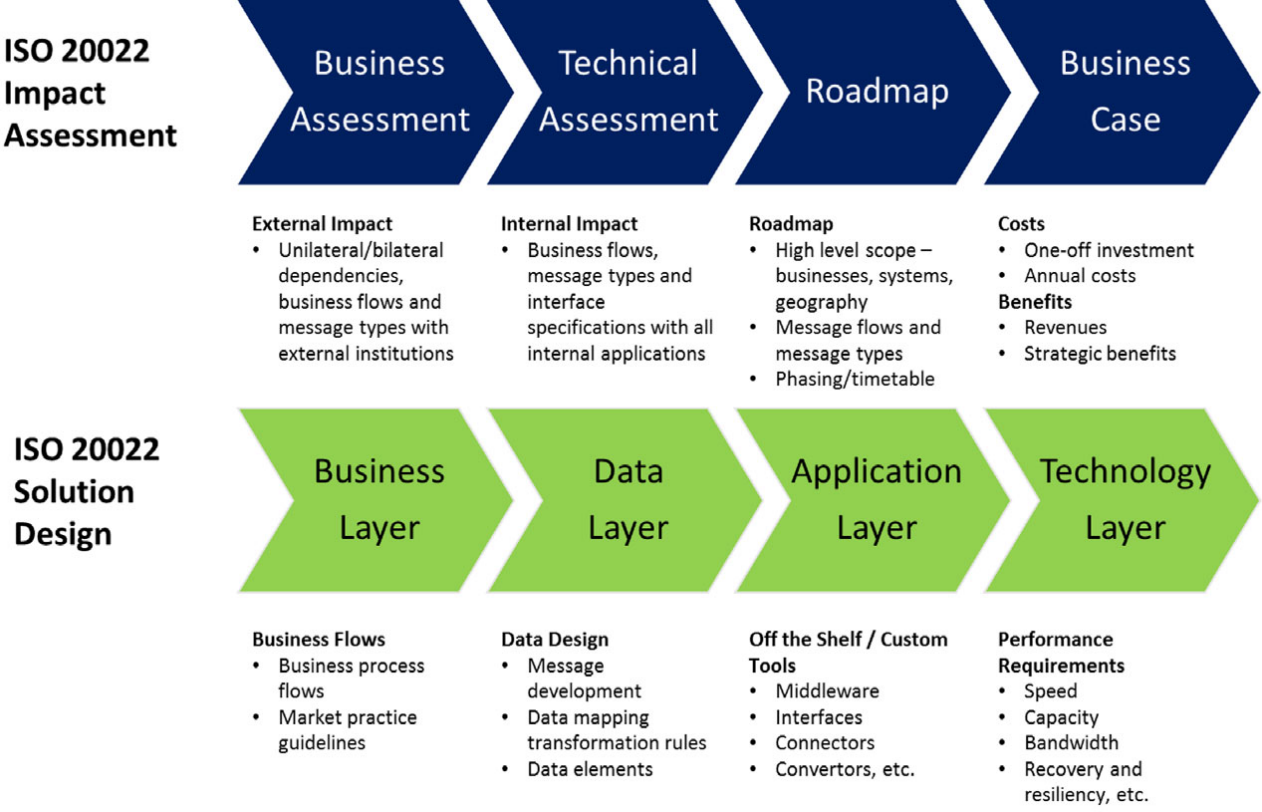
SWIFT ISO 20022 best practice (SWIFT 2015; with permission).

The mainstay of successful projects say that SWIFT (2015, 2016) is how ISO20022 standards ensure that each initiative builds a single, central, and complete view of all data fields, standards, and work flows across all impacted business processes, and their interdependencies. Typically, one of the first tasks would be the “as‐is” and “to‐be” data analysis across existing and future data and business flows. Building a single, central view across all of the data ensures that design, integration, implementation, and on‐going maintenance is accurate, fast, and efficient. The approach promoted by SWIFT to achieve this outcome links the impact assessment and solution design into a seamless process, presented in Figure 3. SWIFT states that this process safeguards the development and implementation of effective and efficient solutions, while ensuring minimum disruption to normal business, by ensuring that business requirements and industry‐level considerations are factored into the development of interoperable solutions. The process also ensures that a cross‐functional holistic perspective guides the development process, which includes ensuring the balanced involvement of third party IT service providers and commercial businesses. Described below, the best‐practice approach championed by SWIFT includes impact assessment and solution design process flows.

Each of the steps shown in Figure 3 is described below. An interesting insight is that the impact assessment and solution design flow in opposite directions in terms of macro in contrast to micro considerations. The impact assessment process first looks at the wider operating environment from a macro level, then steadily narrows its focus down to a micro perspective. Macro‐type questions that would drive the assessment may include: “what gap would the proposed message change fill, or what opportunity would it create compared to the current enabling environment, for which stakeholders and how?” Microtype questions that drive the assessment may include: “why would a business adopt this proposed message change, and in which sub‐sector of the finance industry do such businesses operate?”

In contrast, the solution design process starts with microconsiderations and ends with macroconsiderations. The process begins by assessing intra‐firm considerations, such as “will the proposed change positively impact the current process flows and performance of the target stakeholder; and if so, how?” Subsequent assessments focus on quantifying relationships between inter‐ and intra‐firm considerations, such as “what enablers are required to ensure the proposed benefits are realistic and realizable, along with where and why they presently do or do not exist?”

### ISO 20022 impact assessment

The impact assessment process has 4 distinct steps: business assessment, technical assessment, roadmap, and business case.

#### Business assessment

This stage reviews the firm's current ISO 20022 landscape from an external business perspective, summarizing dependencies, business flows and message types with trading partners (for example, other financial institutions), customers, service providers, and market infrastructures. This means answering:

- What functions will the firm need in order to provide better service or increase efficiency?
- What customer requirements does the firm need to meet, and thus what capabilities does it need to introduce?

#### Technical assessment

This stage reviews the landscape from an internal perspective by summarizing internal data flows and message types, including any new functions that may be required. This means answering:

- What existing applications will be affected? What are the needs, either to produce or to use ISO 20022 data?
- What new capabilities will be required?

#### Roadmap

The Roadmap outlines the future‐state business architecture and general business case for a specific communication change being proposed. Factors considered during the process of establishing the “to be” environment and enabling factors are the scope of the project/investment (including phasing in the new messaging and the expected organizational impact by business area, system, or geography), and addressing any items raised during the impact assessment. The roadmap should list, by phase, impacts on/involvement of:

- external organizations,
- internal business applications, and
- message flows, message types, and versions.

Each impact should be included in the timetable, and then agreed upon by all stakeholders so that the broader interdependencies are fully understood, and the need for action is recognized.

#### Business case

The expected costs and benefits of the proposed roadmap across involved stakeholders and each phase of the proposed change are assessed to develop a business case for the proposed change. Costs included in the assessment are categorized as one‐off and recurring costs. Benefits are categorized as quantified cost savings, incremental revenues, and strategic benefits.

### ISO 20022 solution design

The solution design process also comprises 4 distinct steps/layers: business, data, application, and technology.

#### Business layer

This phase of the design looks at the current design of business process flows and the impact that the proposed change is expected to have on business processes. This phase also determines the guidelines and definitions that need to be considered in the development and eventual communication of the intended change(s).

#### Data layer

This stage of the development process includes examining the extent to which the proposed changes align with the ISO 20022 open business model and the existing ISO 20022 Data Dictionary. Where possible, underlying ISO 20022 data elements will be used in developing the new initiative, thereby helping to ensure its smooth integration alongside current systems and processes.

#### Application layer

The requirements for ensuring the effective and efficient operation of the software and associated tools are assessed. This includes, for example, assessing the impact of the proposed change on existing applications, middleware, interfaces, connections, and converters. The ultimate purpose of the development phase is to ensure harmonization across new and existing applications.

#### Technology layer

The final phase of the development process is to ensure that the networks and processing systems that underlie the successful implementation of the change are adequate in terms of their speed, capacity, bandwidth, and any differing protocol provisions associated with message size or syntax. The scope of this work may extend to the development of recovery and resilience procedures.

### Example of establishing financial interoperability

The following sections build upon the previous descriptions by summarizing the process proposed for establishing interoperable capabilities required to enable a rapidly emerging subsector of the financial industry: A2A. A2A interoperability refers to Mobile Money Operators that provide the ability for customers to transfer funds between 2 accounts at different mobile money schemes, and between mobile money schemes and traditional bank accounts. Mobile Money Operators are used around the globe, including in developing countries where many people do not have access to traditional banks and accounts (Mudita and Deepti 2013; EY 2014).

The implementation of A2A interoperability requires collaboration between commercial companies, who are often competitors. Implementation of A2A also requires collaboration between financial institutions, third party technology solution services, regulators and wider industry stakeholders. The processes proposed by Groupe Speciale Mobile Assn. (GSMA), the global body that represents the interests of mobile telecommunication providers (GSMA 2016), to enable sustainable and fully interoperable A2A systems is described below. The proposed approach reflects that although market requirements and existing infrastructure will differ by jurisdiction, a fundamental need for low‐cost, real‐time solutions applies to every situation.

#### Phase one: establishing strategic priorities

**Figure 4:**
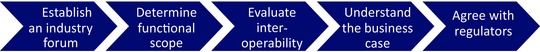
Strategic process to enable A2A interoperability (Clark and Camner 2014; with permission).

The interoperability process begins with establishing dedicated industry groups who are charged with implementing agreed‐upon policies. The 5‐step process proposed by GSMA to identify the pathway required to establish interoperable A2A systems is shown in Figure 4.

**1. Establish industry forum**

The purpose of the forum is to ensure that business, technical, and operational considerations are factored into the strategies, tactics, and operations required to establish interoperable A2A systems. Wherever possible, regulators as well as financial and service providers are represented at the forum.

Nominees must be sufficiently senior, to:

- secure ongoing commitment within their organization across all relevant departments, for example, to participate in design and planning stages, and subsequent implementation, and;
- acquire any necessary resources (budget, information/data, and staff), and resolve significant problems during the project.

**2. Determine functional scope**

Determine high‐level business requirements for the interoperability system in terms of the functionality supported and the objectives of implementation. This takes the form of a due diligence process to identify the requirements and constraints defined by regulations and the commercial concerns of participants, incorporating ISO 20022 methodology described in the previous section.

**3. Evaluate interoperability options**

Identify and agree on the approach that is the most efficient and cost effective for the part of the sector represented.

**Agree on the approach**

Outline agreements for interoperability between firms/supply chains and a collective understanding of the way forward. This includes establishing formal agreements with regulators on technical, operational, and risk management considerations.

**4. Understand the business case**

Once the approach has been agreed upon, the parties involved need to define the commercial details required to produce a business model that is sustainable for all involved parties.

**5. Agree on the approach with regulators**

It is necessary to ensure that the proposed system complies with legal regulations and constraints. Therefore prior to implementing the strategy and the tasks required to achieve the strategic intents that lie behind an initiative (formalized during the business case assessment, at which point it is ratified with industry participants), the commercial and technologic pathway for rolling out the interoperability enablers and proposed A2A systems will be finalized with the regulator(s).

#### Phase 2: Implementing strategy

**Figure 5:**
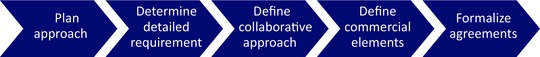
Implementation pathway (Clark and Camner 2014; with permission).

Figure 5 shows the 5‐step process that GSMA developed for implementing an interoperability rollout strategy. Each step has 2 workstreams: technical, followed by operational/commercial. Technical workstreams define the technical implementation for the chosen option, the technical service‐level requirements, and the design of the standard interfaces that need to be created. Operational/commercial workstreams define target‐pricing models, operational procedures, such as fraud/risk mitigation and customer care, and the formal agreement between the participants, including service‐level agreements.

Conducting the 2 workstreams in parallel lessens the likelihood that unexpected issues may arise which could limit the effectiveness and efficiency of the resulting system or markedly increase the costs associated with the development, implementation, or operation of the system. Unexpected issues could include the extent to which a developed system is able to interact with more extensive systems, whether they are regional or global, versus what was envisioned and is necessary to establish a commercial, sustainable service.

**1. Plan approach**

The Task Force that will oversee implementation of the strategy is formalized by participating organizations who formally sign‐off on the strategy and express their commitment to continue. Translating strategy into action requires the Task Force to define a critical path that encompasses anticipated milestones (activities and deliverables), collaboration requirements, and the anticipated resources required to complete the implementation process. The resulting document will be disseminated within and between the participating companies, with the proviso that it is a working document that will evolve as the planning and implementation process proceeds forward.

The criteria against which GSMA recommends that implementation options and technical considerations are scored include:

- Risk impact
- Implementation complexity
- Transaction cost impact
- Regulatory framework
- Agreement framework
- Scalability
- User experience
- Time to market

**2. Define requirements**

Once agreement is reached, business requirements and participants’ functional and service level requirements are defined in detail. These will feed into both technical and operational workstreams, and allow traceability and reference points for acceptance testing of the implemented solution to be established. The internal resources that each organization must commit to the implementation plan at each stage of the implementation process are also defined and understood, along with the commitments and expectations required from specific members of the Task Force.

**3. Define collaborative approach**

Stakeholder collaboration is separated into 3 categories of work, each of which are overseen by the Task Force.

Collaborate to define standard interfaces

To minimize implementation and operational costs, interoperability should be implemented using an agreed‐upon standardized approach; and interface specifications should be based on international standards where appropriate. A key exercise for the Task Force is to develop and agree on appropriate technical interface specifications.

**Define collaboration for operational procedures**

Define cross‐organization operational procedures that need to be aligned to ensure that data can be reconciled across organizations for all transfers, successful or otherwise.

**Collaborate on risk mitigation**

It is imperative that risks are understood and can be managed effectively. A key activity for the Task Force is to develop an understanding of potential risks and their level of exposure, then to recommend appropriate mitigation policies and procedures for operational services to follow. This may lead to a formalized risk‐management framework for the industry, and may foster use of best practices among participants.

**4. Define commercial elements**

Elements of the commercial agreements that must exist between participating organizations and stakeholders are defined. Depending on the implementation option selected these agreements may need to be collaborative; examples are agreement on fee structures and sharing of data.

**5. Formalize agreements**

Formal agreements on the collaborative aspects, along with the individual roles and responsibilities of organizations, as defined and agreed upon by the Task Force and participating stakeholders, will be signed. Having defined technical and operational considerations associated with each of their financial supply chains, the development and implementation process commences in earnest. The activities and outcomes detailed in the rollout strategy and plan, including regular communications with stakeholders, are overseen by the Task Force.

## Travel Industry

The scale, geographic location, and capabilities of businesses that operate in the travel industry differ enormously. The regulations under which these businesses operate differ immensely as well, by sector (for example, airlines in contrast to hotels) and by jurisdiction (for example, Indonesia in contrast to Germany). A challenge in implementing interoperable systems in the travel industry is that many of the commercial transactions conducted by businesses occur by fax rather than by computers, especially among small independent operators located in developing regions with limited access to modern IT capability and infrastructure.

The OpenTravel Alliance (OTA) was established to “provide a community where companies in the electronic distribution supply chain work together to create an accepted structure for electronic messages, enabling suppliers and distributors to speak the same interoperability language, trading partner to trading partner (OpenTravel 2011). The OTA is a member‐funded non‐profit organization formed in 1999 by major airlines, hoteliers, car rental companies, and service providers that distributes data and provides technology systems to the travel industry. While members are the primary focus of the OTA's activities, the protocols and systems that it develops through a consultative process are shared with the wider industry. The decision to adopt these protocols and systems rests with individual businesses (Open Travel Alliance, undated; Perini 2007).

The OTA's primary activity is to develop and maintain a library of XML schemas for use by the travel industry. Schemas are a set of rules to which XML documents, for example, must conform. The OTA also:

- Administers the register of firms implementing OpenTravel specifications and provides guidance on the XML architecture;
- Creates open messaging specifications in XML for every vertical in the travel industry; promotes the use of those messages and provides implementation guidance, and
- Hosts an annual conference—The Advisory Forum.

### Governance

The governance model established to manage the interoperability capabilities, including communication protocols and schemas, that the OTA was established to oversee is shown on pages 1 to 13 of the OpenTravel Implementation Guide (OpenTravel 2010). The OTA has a 4‐tiered governance model that is managed by a small team of staff.

The model consists of:

- Board of directors
- Interoperability committee
  ○ Data content/best practice subcommittee
  ○ Marketing subcommittee
- Working groups
- Ad hoc project teams

#### Board of directors

The Board of Directors is comprised of a representative from each industry involved and is elected by the membership. Industries represented by the board include airlines, car rental firms, hotels, cruise lines, railways, leisure suppliers, service providers, tour operators, travel agencies, solutions providers, technology companies, and distributors.

The role of the board includes:

- Contacting OTA staff, which includes an Executive Director, specification managers, and technical administrators;
- Overseeing the governance process, to develop and communicate messaging protocols and standards; and
- Providing strategic guidance on the OTA's role and value proposition to members.

#### Interoperability committee

The Interoperability Committee consists of elected representatives from each of 4 vertical working groups that it oversees. The committee also works closely with 2 subcommittees: data content/best practice, and marketing. Any OTA member may participate in a committee, working group or project team, all of which are volunteer positions.

The primary role of the committee is to:

- Ensure consistency across working group efforts and outcomes, including the development of effective interoperability solutions/messaging;
- Provide a conduit between the Board, OTA staff, industry, subcommittees, and solution providers; and
- Oversee the ad hoc project teams, which may, for example, work on development of a specific new message format or protocol for enabling interoperability.

Subcommittees:

- Data content/best practice: reviews all XML schemas proposed by working groups and ad hoc project teams.
- Communications: provides strategic and engagement support on marketing and communications.

#### Working groups

The activities of each working group relate to a specific topic that is considered key to enabling and beneficial for the implementation of interoperable solutions. Working groups also form the first step in the process of assessing the validity and potential value of new messages or schemas proposed by OTA members.

There are 4 permanent working groups (transport, hospitality, architecture, and travel integration). If it is deemed that one of these working groups does not have the necessary knowledge, skills, or capabilities to address a topic or issue, an *ad hoc* project team will be established. The most likely reason for establishing an ad hoc project team is to oversee the development of a new message format, protocol, schema, or interoperable solution.

### Development of new specifications

The OTA operates open standards, which are free. New or revised schemas are issued as specifications twice a year, along with user guides. The OTA believes that this zero‐cost model spurs adoption, and widespread adoption generates wider implementation and further interest in specifications, which in turn drives membership, providing the Alliance with income.

This approach enables members and non‐members to download free OpenTravel schema, including XML files, XSD (EML Schema Definition) files, the 700‐page User Guide, the full code table, flattened schema files, and Best Practice documents. An open access forum (http://www.opentravelcommunityforum.com/forum/) enables non‐members to access general discussions on implementation and documentation.

Benefits, accessible only to OTA members, include:

- Access to the specifications manager, OpenTravel wiki, and full Implementation Guide;
- Detailed discussions on architecture, hospitality, transport, travel services, tours, and OTA activities;
- Annual advisory forum attendance;
- Submission of a project team proposal (PTPs);
- Working group mailings;
- Participation in working groups and access to meeting minutes;
- Documents, protocols, schema in development; and
- Registering the implementation of software, use of open source schemas.

The process for developing new schema is described below.

#### Schema development process

The OTA has established a structured 5‐step governance and engagement process for developing new schema, with different committees and stakeholders involved at specific stages of the process. The process followed in the development and release of OTA specifications, which typically takes 6 mo from start to completion and publication, is presented on pages 1 to 14 of the OpenTravel Implementation Guide. The 5 steps, summarized below, are:

- Project team proposals (PTP)
- Development
- Member review
- Public review
- Final publication

The OpenTravel Developers Network website (OpenTravel Alliance 2014) provides a single portal where both members and non‐members can learn about and download new schema, access technical support for implementation purposes, and provide feedback on specific schema.

#### Project team proposals

Any member may propose a PTP by stating the purpose, scope, and resource requirements. The most relevant work group members review the proposal, which must then be approved by the Interoperability Committee (IO).

There are three types of PTPs:

- Business requirement definition (no schema work)
- New schema
- General projects (for example, a study)

Once approved for further development, the group to which the work has been assigned determines the timetable, including milestones and deliverables. The OTA then notifies the submitting member that their submission has been accepted, and conveys key milestones targeted for development of the schema, including intended completion date.

#### Development of new schema

From the initial PTP, and based on the alliance's guidelines, project teams create a schema which work groups review and approve as a draft. The quality of the draft schema is then assured by the data content/best practice subcommittee. If approved, the draft schema is submitted to the IO for member review. If not approved, the draft schema is returned to the project team for revision, before being resubmitted to the data content/best practice subcommittee.

#### Member review

The member review is a 30‐d comment period, for members only. Member feedback is resolved by project teams and work groups, before the schema is re‐submitted to the data content/best practice subcommittee, and then the IO committee to approve before being issued for public review.

#### Public review

The full public review allows a 30‐d comment period for both members and non‐members, with the same subsequent feedback and resolution process and approval as occurs during the member review. The final step in development of a new schema is finalization of the User Guide, which includes sample use cases to assist industry in acting on the proposed changes.

#### Publication

The schema and associated materials are published, along with the user guide. Avenues through which the schema and supporting materials are published include OTA's forum and blog, OTA member newsletters, OTA's annual conference, and industry publications.

### Example of implementing interoperability

The OTA has been criticized for not serving the needs of smaller operators, as its approaches lead to outcomes that are more suited to assisting larger operators in benefiting from interoperability, partly because they have greater scope and economies of scale. Regardless of size, member participation in the OTA is critical to deriving value from membership.

Members that have benefited the most from OTA's activities include those that have joined as a cohesive group (Nayar & Beldona 2010). For example, Norwegian Cruise Line (NCL) wanted to communicate with multiple distribution partners using the same schema. So the NCL joined the Alliance as its first cruise member, and recruited another cruise line, a solutions provider, and 2 technology providers. The organizations then together mapped out a set of messages needed to facilitate electronic distribution of complex cruise functions. This resulted in their ability to reduce operating costs and improve cruise passenger satisfaction rates, likely resulting in market growth and increased revenue.

For hotels, it is generally agreed that the costs of implementing OTA standards were high at the outset, but that there was direct return from increased yield per room subsequent to implementation. This speaks to the benefits of interoperability, and that the value proposition of interoperability can be both transparent and readily validated.

While the OTA's approach to enabling interoperability may not benefit smaller and larger operations equally, evidence exists that its approach does not automatically preclude smaller operators or service providers from benefiting from the open‐sourced XML schema. Examples include how adopting open standards enhances the ability of intermediaries (processors, in the seafood industry context) to access more suppliers irrespective of the technology platforms that suppliers use, thereby offering customers a greater choice of products.

Three further insights are worth noting. The first is that the OTA needed to establish more links with other industry bodies and the American Natl. Standards Institute (ANSI) before it was able to ensure that best practice standards were incorporated into the development and implementation of schema. Second, while no initial investment in hardware and software was required for OTA to enable interoperability, external help was necessary to implement specifications in the first instance. Subsequent upgrades and further deployments were completed by in‐house resources. Third, while motivations of implementers to use the open‐sourced XML included enhanced ability to add/delete channels without incurring the costs of building, operating, and maintaining proprietary connections, industry‐wide interoperability may actually reduce commitment between business partners along supply chains because there is less cost/risk involved in switching.

## Produce Industry

The fresh fruit and vegetable industry is a global, high‐value industry supplying a wide range of products and generating approximately $2 trillion in annual revenue (First Research 2015). The industry is fragmented with more than 200,000 businesses in the United States alone. The extent of the size range of businesses involved in the U.S. produce industry is illustrated by the 50 largest wholesale firms accounting for approximately 30% of total revenue, which for the entire industry was estimated at $122.1 billion in 2010 (Cook 2011; First Research 2015). Many types of suppliers, distributors, wholesalers, shippers, and importers, work with food service operators and food retailers, making industry‐wide generalizations difficult, particularly because company characteristics tend to vary by the product or product group that each sector supplies (Cook 2011). Two major current trends in the industry are the growth of sales direct from farm to consumer, and growing imports of fresh produce from developing countries. In 2010, imports of fresh produce into the United States were valued at $12.3 billion (Cook 2011).

All fresh produce shares 2 characteristics: (1) perishability, which limits storability and (2) seasonality, which creates supply challenges and drives imports (Cook 2011). Characteristics that differ by produce type, the specific operations of businesses, and the market supplied include: (1) marketing efforts that lead retailers, foodservice operators, and their suppliers to expand their range of products and packaging formats; and (2) aggregation and/or subsequent disaggregation of products as they move along the supply chain. Coupled with increasing consumer demand for year‐round, high‐quality, fresh produce, and the introduction of mandatory food safety traceability requirements (for example, the U.S. Food Safety Modernization Act), the challenges of implementing traceability in what essentially remains a commodity industry are significant. Because seafood has characteristics similar to those of produce, the lessons that can be learned from the development of full chain interoperability in this sector are particularly valuable.

The PTI was the first industry‐led commodity‐wide traceability effort in the United States, which was created in response to a 2006 U.S. food safety incident and product recall stemming from organic spinach unknowingly contaminated with *Escherichia coli 0157:H7* (Treacy, unpublished personal communication). Inability to quickly identify the source and then recall contaminated product resulted in the FDA issuing a notice to “not eat bagged fresh spinach” and consumers losing confidence in leafy green vegetables. By 2007, losses resulting from the incident, which had taken many months to rectify, were estimated to have reached US$350 million, with sales reaching just 80% of pre‐recall levels, and the failure of many previously viable businesses. This, along with more than 200 illnesses and 4 deaths after consuming contaminated spinach, created an impetus for the U.S. produce industry to implement full chain traceability (Treacy, unpublished personal communication; Treacy 2016). While businesses operating in the produce industry had good internal traceability, albeit ranging from predominantly paper‐based to largely electronic, the key weakness was in lack of traceability between businesses.

### Governance

**Figure 6:**
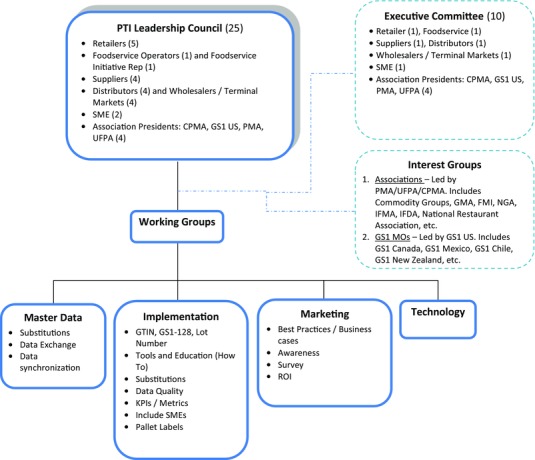
Produce traceability initiative governance structure (PTI 2010; with permission).

The governance system of the PTI organization, which was formed in February, 2010 and originally comprised of 5 interdependent groups, is shown in Figure 6. A sixth group (Buyers Working Group) was formed later, in 2013. The 6 PTI groups are:

- Leadership council
- Master data working group
- Implementation working group
- Industry communications working group
- Technology working group
- Buyers working group (not shown in diagram)

#### Leadership council

The PTI Leadership Council acts as a Board of Directors that meets twice a year face‐to‐face and participates in monthly conference calls. The Council is comprised of influential senior representatives from industry, including CEO's and company executives, and an Executive Committee that communicates via bi‐weekly conference calls. The Executive Committee determines the Leadership Council's meeting agendas, guides discussions, and conducts first reviews of issues raised by working groups.

The Leadership Council:

- Sets the strategic direction of the PTI organization and addresses barriers to implementation.
- Provides oversight for project scope and timetables.
- Assigns issues to working groups, and monitors working group execution and performance.
- Monitors progress in meeting milestones, stakeholder commitments, and industry adoption.
- Serves as spokespeople with news media and industry.
- Ensures appropriate dialogue with other industry associations, stakeholders, and similar industry initiatives.

Ensuring effective 2‐way dialogue with industry stakeholders is a basis for the formation of the interest groups identified in Figure 6. Interest group members range from sector advocacy organizations (for example United Fresh Produce Assn.) to other key stakeholders (for example country‐based GS1), who are invited to attend meetings as a means of engaging with the industry at a grassroots level. Engaging GS1 and the Canadian Produce Marketing Assn. from early on in the initiative has helped ensure that while the focus of the organization is primarily the United States, its operations incorporate the needs of businesses involved in importing produce into the United States.

#### Implementation working group

The Implementation Working Group monitors, guides, and promotes voluntary adoption of GS1 standards that align with milestones and objectives determined by the other working groups and the Leadership Council. Ensuring development and implementation of appropriate common industry standards upholds the synergistic operation of the standardized electronic PTI traceability system with other functions of businesses, such as accounting and logistics, and simplifies adoption by businesses regardless of their size, location, or products handled. Specific Implementation Working Group roles and objectives include:

- Develop template for industry adoption of the PTI standardized electronic traceability system by all trading partners, from growers to retailers;
- Develop solutions to address implementation issues;
- Identify and promote best practices through development of implementation tools;
- Define, lead, and facilitate PTI pilots; and
- Develop measurement tools and metrics for the macro overall initiative and at the micro individual business levels, to encourage further adoption of the PTI traceability system and guide continual improvements in performance.

#### Technology working group

The Technology Working Group provides an open forum where third party technology providers can regularly discuss strategies and adoption processes, with the primary purpose of accelerating industry adoption of the PTI traceability system and continually improving performance. This is achieved through working group focus on application of GS1 standards‐based solutions to businesses operating along the entire supply chain, from growers to retailers and key input providers such as label manufacturers. Specific Technology Working Group roles and objectives include:

- Educate technology providers serving the produce industry about GS1 standards and the PTI system
- Provide a forum for businesses that have adopted the PTI system to collaborate with technology; providers, to successfully drive implementation and continually improve performance; and
- Develop solutions for businesses of all sizes, locations, and technical capabilities.

#### Master data working group

The master data working group determines the most appropriate means for identifying produce attributes and communicating that data between trading partners using GS1 standards and the GS1 Global Data Synchronization Network (GDSN). Specific master data working group roles and objectives include:

- Address and develop options for product substitution,
- Provide best practice options for data exchange and data storage, and
- Provide best practice options for synchronizing data exchanged between trading partners and appropriate industry stakeholders.

#### Industry communications working group

The Industry Communications Working Group accelerates adoption of the PTI standardized electronic traceability system through the facilitation of discussions on communication strategies and support of activities designed to accelerate the adoption of PTI best practices. This is achieved through ensuring accurate and effective communications, securing spokespersons, serving as subject matter experts, and generating business case studies to support the adoption of best practices by individual businesses along the supply chain. Specific Industry Communications Working Group roles and objectives include:

- Develop, review, and approve marketing and communication strategies.
- Review and approve marketing materials and efforts to drive awareness of the PTI.
- Drive adoption of the PTI traceability system and implementation of best practices through sharing of produce industry business cases.
- Engage in consistent messaging to communicate PTI highlights and progress in meeting performance targets from macro initiative‐level and micro business‐level perspectives.
- Report on scorecard metrics and related industry initiatives to the Leadership Council.

#### Buyers working group

More informal than the other working groups, the buyers working group provides a forum where retailers and foodservice operators can discuss issues that impact their adoption of the PTI traceability system, which differ from those impacting suppliers’ decisions. This group was formed following the realization that expectations communicated early in the development of PTI were not required to ensure effective full chain traceability. For example, it was found that scanning each item when picking deliveries at retailers’ distribution centers prior to shipping to individual stores, as proposed early in the initiative's development, did not provide a sufficient cost benefit to make it worthwhile and encourage widespread adoption.

The most important point for traceability purposes in retail or foodservice is at the level of individual stores and restaurants, in the knowledge that the option remains to enact traceability at distribution if required—such as during a recall. However, when suppliers learned that individual items were not scanned at retailers’ distribution centers, some questioned the value of PTI and retailers’ commitment to its implementation. This created adoption issues that could have been avoided had retail and foodservice buyers been more closely involved in PTI's early development. Specific Buyers Working Group roles and objectives include:

- Identify and communicate best practices in retail and foodservice operations
- Encourage adoption of the PTI traceability system and GS1 protocols across corporate and functional operations Contribute to business case studies in collaboration with suppliers, and
- Report to working groups and the leadership council the performance metrics and issues found to impact adoption or attainment of best practices.

### PTI's evolution

The PTI began with the realization that the internal processes and identification systems developed by individual businesses, often in isolation of business partners and industry groups, were a primary reason for the major economic and social crisis that had befallen in the industry in 2006. However accurate businesses were in tracing products internally, lack of interoperability meant that products could not be tracked as they moved along the supply chain between trading partners. This lengthened the time required to gather the information required to correctly identify products and prevent contaminated items from reaching the market. Lack of interoperability also led to operational inefficiencies. Many products were sold unpackaged at retail, preventing the application of a universal product code which would assist investigators in identifying products in the event of a recall, exacerbating the challenges faced during the spinach crisis. The initial impetus to establish the PTI began with the Board of Directors from 3 key produce industry organizations recognizing a need to implement an effective full chain traceability solution following the spinach crisis. The effectiveness of such a system relied on developing a common approach that was simple and efficient and which could be adopted by businesses whose technological capabilities ranged from professional standalone IT departments with enormous resources to individual farmers with a laptop and printer.

The 3 organizations that first sponsored the initiative and made traceability a priority were the Produce Marketing Assn. (PMA), Canadian Produce Marketing Assn. (CPMA), and United Fresh Produce Assn. (UFPA). Together with invited organizations who included the Food Marketing Institute, Canadian Council of Grocery Distributors, Canadian Horticultural Council, Intl. Foodservice Distributors Assn., and Natl. Restaurant Assn., a Steering Committee and Action Plan were created. The Steering Committee comprised respected individuals chosen for their experience and attitude, who were sufficiently senior to address challenges to implementation that arose in their own businesses or the wider industry.

Launched in October 2008, the PTI Action Plan comprised 4 key elements, shown in Table 1. The plan's finalization and proposed implementation timeline, with 34 businesses committing to embrace a standardized electronic traceability system based on batch/lot level barcoding by December 2012, was spurred on by another food safety incident, in 2008, which affected more than 1400 consumers. It took 3 mo to identify the source as fresh jalapeño and serrano peppers from Mexico.

**Table 1:** Foundational elements of produce traceability initiative (PTI) (Treacy 2012, 2016a; with permission)

Element of PTI action plan	Description
Mandate GS1 standards	GS1 traceability standards, established in 2004, based on standards developed by the Canadian industry collaborative initiative Can‐Trace to achieve system‐wide internal and external food traceability.
GS1 standards timeline	Steering Committee agreement on developing formal timeline for adoption of GS1 standards, by first establishing time required to adopt GS1 standards within own operations.
Show support and commitment	Determination that the best way for businesses to show their support and commitment to PTI was for only the Produce Marketing Assn., United Fresh Produce Assn., and Canadian Produce Marketing Assn. members that agreed to abide by GS1 standards to participate in the Leadership Council or Working Groups.
Case‐level traceability forms the backbone of traceability	Case‐level traceability set as the common standard because the vast majority of businesses already recorded Batch/Lot Numbers. Definition of PTI case level is cases, cartons, boxes, flats, returnable plastic containers (RPCs) and bins.

The foundational elements listed in Table 1 allowed the implementation of a standardized traceability system that did not require businesses to share data or information beyond a global trade item number (GTIN) and a batch/lot number (BLN) contained in a GS1‐128 barcode. Businesses that wish to share additional data, such as advance shipping notices, or directly link their computerized systems for more advanced collaborative purposes, can do so in conjunction with the PTI traceability system.

Virtually all produce businesses were recording case‐level movements, thereby making it the preferred choice for traceability purposes, as a result of the introduction of the U.S. Bioterrorism Act of 2002, which requires each handler of food products to keep records documenting the movements of its products 1‐step forward and 1‐step back in the supply chain. This requirement led most businesses to develop an internal traceability system. The PTI system provided the means to establish external traceability in the industry. This was achieved by building on businesses’ internal traceability systems in 2 ways: (1) establishing a common nomenclature for product identification using the GTIN as a common numerical identification system for each product, and (2) requiring that each firm track 2 common pieces of information (the GTIN and BLN) as each case of produce moves through the supply chain. This system is facilitated by the fact that every firm in the supply chain handles a standardized unit of product – the shipping ‘case’ (PTI 2011). That the definition of a case differs according to individual arrangements agreed upon between trading partners, reflects the simplicity and flexibility that belies PTI's success. For example, so long as they abide by common standards and protocols, individual businesses determine how they operationalize PTI. Within 7 y of the beginning of the program, this combination of factors led to approximately 60% of all produce shipped in or out of the United States carrying a PTI label (Treacy, unpublished personal communication). Preventing the need to mandate that businesses surrender control of their data also proved key to PTI's success.

**Figure 7:**
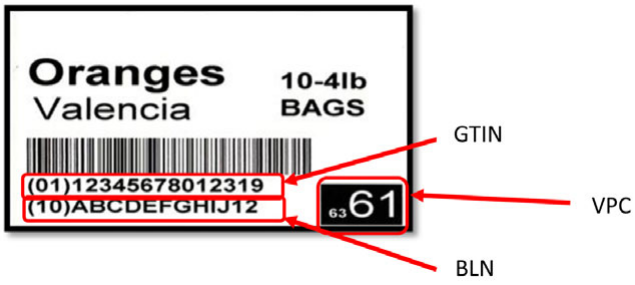
Standardized GS1 verified label (Treacy 2012; with permission).

For use in distribution centers equipped with advanced logistics technology, the label incorporates a CRC‐16 Hash Computation algorithm that translates the GTIN and Lot Number into a 4‐digit number known as the PTI Voice Pick Code (VPC). The VPC is read into a voice‐directed picking system that accurately assigns GTIN and Lot Number to the case picked. Figure 7 shows the first standardized label developed by the PTI Working Groups for use on all cartons, boxes, and containers, except returnable plastic containers (RPCs). Other labels developed include those required for RPCs and pallets/flats.

The implementation timelines developed by the PTI Steering Committee are shown in Table 2. The development and assigning of company prefixes, GTINs, VPCs, and barcodes is performed by GS1, with information and support accessible online to PTI participants.

**Table 2:** PTI milestones established by steering committee (Treacy 2016a, 2016b; with permission)

Milestone	Target completion date
Obtain a company prefix from GS1	2009
Assign GTINs to cases	2009
Provide GTIN information to buyers	2009
Show human readable information on cases	2011
Encode information in a barcode	2011
Read and store information on inbound cases	2011
Read and store information on outbound cases	2012

A tragic food safety incident in 2011 that involved cantaloupes contaminated with listeria which caused 33 deaths effectively shut down the U.S. cantaloupe industry, further motivating businesses to embrace the PTI traceability system. The severity of the incident could have been considerably less if the entire chain had been PTI‐system compliant (Treacy 2016).

### Technical standards

The most important practices pertaining to the PTI standardized electronic traceability system is adherence to common GS1 protocols to establish common nomenclatures for products, trading parties, and individual cases, along with a standardized means of communicating that information in 3 ways: machine readable, human readable, and voice pick. This process is managed by GS1. The use of existing standards allowed businesses to adopt the PTI system without making wholesale changes to their operations, which would have delayed introduction of the PTI system and led to industry resistance. Participation of country‐level GS1 organizations (for example, GS1 New Zealand) in the working groups and interest groups ensures that the same standards are applied to domestically‐produced and imported products. Table 3 lists the 7 milestones created by the Steering Committee during development of the PTI Action Plan, to assist the effective and efficient implementation of GS1 standards and protocols. The milestones are listed in the order that they must be achieved to ensure the successful implementation of PTI systems. Closely resembling the implementation timelines contained in Table 2, the implementation milestones establish individual responsibilities and accountabilities for ensuring effective full chain traceability.

**Table 3:** Milestones to guid1e implementation of GS1 standards and protocols (Treacy 2016a; with permission)

Number	Milestone description
1	Brand owners (suppliers) obtain a GS1‐issued company prefix
2	Brand owners must assign 14‐digit global trade item numbers (GTINs) to all case configurations. A number assignment strategy minimizes the number of GTINs created and ensures consistency across industry segments.
3	Brand owners must provide and maintain their GTIN information (and corresponding data) to their buyers.
4	All parties must have the systems required to capture and store GTINs, and subsequent information.
5	Those packing the product are responsible for providing the GTIN, lot number, and pack/harvest date in a human‐readable form on each case.
6	Those parties packing the product are responsible for encoding the GTIN, the batch/lot number, and the pack/harvest date in a GS1‐128 barcode.
7	Each handler of the case must read and store information both 1 step up and 1 step down the supply chain: GTIN, batch/lot number, pack/harvest date, shipper ID, shipper name, shipper address, receiver ID, receiver name, receiver address, date of shipment, date of receipt, quantity, unit of measure, and shipment ID.

PTI's Leadership Council and working groups do not determine which technologies trading partners should implement. Neither does it promote specific hardware or software solutions, beyond stating that technologies should be GS1 compliant, which they typically are as a result of GS1 being the “global language of business” in most industries (GS1 2016). The working group arrangements ensure that while technology solution providers have a voice in PTI's development and management, decisions are based strictly on users’ perspectives, including those of retailers whose operations are typically the most complex of the involved stakeholders. Working groups are co‐chaired by an industry representative and a staff member from 1 of the 4 associations that administers the PTI system. Technology solution providers and consultants can only participate in the Technology Working Group. This ensures that all technical or standard‐related decisions are nonpartisan and cannot be influenced by stakeholders’ size or market power. Members of the Leadership Council and working groups may recommend various hardware and software options to others, though strictly on an informal basis. Decisions on PTI label format and mandatory information contained on the label are made by the Leadership Council based on recommendations from the working groups, not individual members.

### Implementation

**Figure 8:**
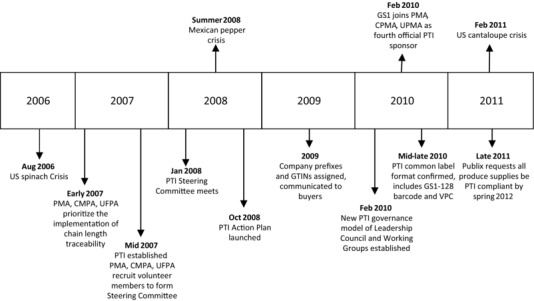
Timeline of PTI's implementation (Treacy 2012, 2016a; with permission).

The process of implementing the PTI system essentially began when each of the Steering Committee members that had been recruited by PMA, CPMA, and UFPA began work to determine how long it would take to implement GS1 standards in their own business. The 45‐member Steering Committee—comprising 6 foodservice companies, 11 grocery retailers, 20 produce suppliers, along with representatives from PMA, CPMA, and UFPA—first met in January, 2007. A timeline of key milestones achieved during the evolution and implementation of the PTI system is shown in Figure 8.

The timeline shows the activities and outcomes that formed the initiative's foundation and led to it becoming the recognized U.S. produce industry standard for traceability. Publix (Lakeland, Fla) was the first retailer to request that suppliers become PTI‐system compliant by the spring of 2012. This was followed in 2013 by Walmart (Bentonville, Ark) and Whole Foods Market (Austin, Tex). Other U.S. and Canadian retailers have or are working towards adopting the PTI system as their traceability standard for fresh produce. Foodservice operators have also adopted the PTI system.

Lessons learned from case studies and pilot projects led to the development of materials and tools for businesses to follow during implementation. They have also both enabled and informed revisions to GS1 global traceability standards (GS1 2012). The development of the 7 best practice milestones and other materials, along with continual improvement of PTI‐system capabilities, have been enabled by the strong governance system implemented in 2008. Materials available through PTI's website reflect the Leadership Council's pragmatic unswerving focus on developing traceability solutions that are simple, practical, and which use existing capabilities and resources wherever possible by actively engaging with all levels of industry. A critical element of PTI's governance model is the purposeful engagement of only likeminded individuals who have a track record of implementing solutions in challenging situations and who are respected by industry.

Businesses along the entire supply chain have benefited from implementing the PTI traceability system, with specific examples presented in PTI case studies (PTI 2016). A benefit common to all businesses is the ability to conduct faster, more streamlined, and less costly recalls. Benefits achieved by retailers include reduced labour costs, faster and more accurate distribution, and more efficient operations. The PTI system has reduced labor costs in stores and enabled more effective inventory management, which retailers, including Walmart (Webber 2014), say has improved quality and reduced shrink. Adoption of the PTI system also enables retailers to reduce the likelihood of litigation, instigated by government and private parties with increasing regularity following food safety incidences.

Other stakeholders that have benefited from implementation of the PTI system include foodservice distributors. Implementation allowed improvements in their inventory management practices and helped them to connect traceability with accounting or other functions. Beyond labor savings, PTI system implementation has also resulted in more effective invoicing and accounting practices. Growers and shippers, such as Frontera (Edinburg, Tex) have benefited from increased visibility along the supply chain enabling them to make more informed management decisions, resulting in the ability to reduce costs and increase revenues through improved quality management practices. The distributor Charlie's Produce (Seattle, Wash., U.S.A.) achieved significant financial gains by using the PTI system to improve inventory and distribution management practices. The system, however, does not provide traceability at the unit level.

## Healthcare

In healthcare, interoperability is the ability of different IT systems and software applications to communicate, exchange, and use data among clinicians, laboratories, hospitals, pharmacies, and patients regardless of the application or application vendor. While the opportunities that interoperability offers the healthcare industry by enabling more cost‐effective treatment and improved patient care are significant, the lack of an overarching governance model such as that which exists in the finance industry (Gamble 2012) and to a lesser degree the travel industry is impacting the rate at which interoperability is enabling those opportunities to be realized.

Other barriers to establishing effective and efficient interoperability in health care include the fact that the industry is viewed from a national or regional perspective, rather than an international one. Researchers (for example the Bipartisan Policy Center 2015) say that the existence of multiple stakeholders with competing interests combined with a lack of common standards has also slowed the pace of interoperability, which led to the lack of a clear business case for why healthcare should adopt interoperability. This has also led to developments and activities occurring at a regional or institutional level. It has also led to most interoperability developments occurring within individual stakeholder groups (for example between physicians and/or hospitals), not between differing stakeholder groups (for example between physicians and medication allergy centers). This has led to interoperability initiatives differing in their approach, resulting in relatively isolated examples of organic incremental change versus the rapid change in interoperability practices and capabilities experienced in previously described industries.

### U.S. healthcare sector

**Figure 9:**
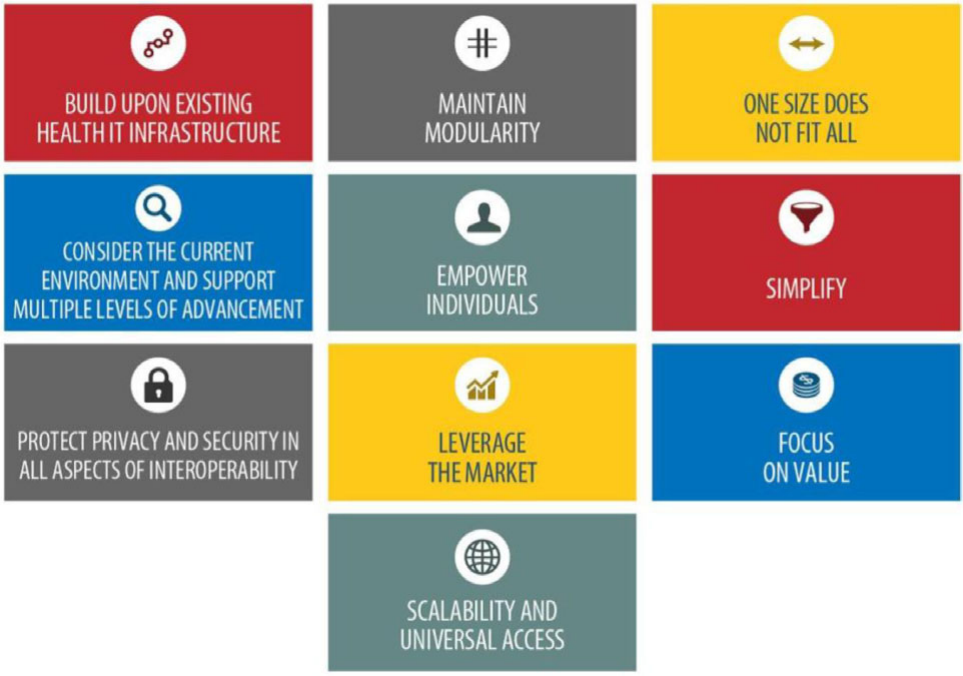
Principles of interoperability in U.S. healthcare (NCHIT 2015; with permission).

Figure 9 shows how the U.S. Office of the Natl. Coordinator for Health Information Technology (NCHIT 2015) views the principles that could allow quick wins to be made within a long‐term vision of enabling interoperable communication and information flow.

The principle of U.S. healthcare interoperability set out by NCHIT and other research organizations primarily falls into 4 categories (governance, policy, operations, and standards), each of which are summarized below:

#### Governance

**Figure 10:**
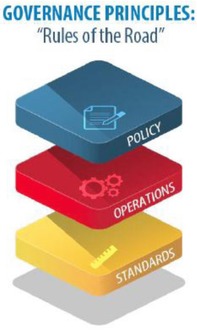
Rules of the road governance principles (NCHIT 2015; with permission).

The objectives of an effective governance system for the U.S. healthcare industry would be to establish an enabling environment that is conducive to the broad development and adoption of interoperable capabilities. The NCHIT indicates that the governance process would itself be guided by the 3 “Rules of the Road” principles (NCHIT 2015) that are presented in Figure 10. Each rules of the road principle follows.

Applying these Rules of the Road would assist the organization(s) that oversee(s) the governance process to readily and efficiently:

- Identify common policies, operational or business practices, and standards to support services that enable interoperability.
- Provide a mechanism for establishing trust across trading partners, that is, confidence in the practices of the other people/organizations with whom electronic information is shared. This acknowledges that while trust can be established among specific, known groups of trading partners through local governance, data‐use agreements and other contractual arrangements, it is also be important to have mechanisms for scaling trust beyond such known groups. This requires assurance that each data holder adhered to a set of common policies, operational and/or business practices, and technical standards.
- Enable collective decisions between competing policies, strategies, and standards in a manner that does not limit competition.
- Coordinate ongoing collaborative decision‐making about enhancing interoperability.

Although not all of these Rules of the Road may be relevant/transferrable to the seafood sector, they give a useful checklist of the types of principles which need to be established upfront.

**Figure 11:**
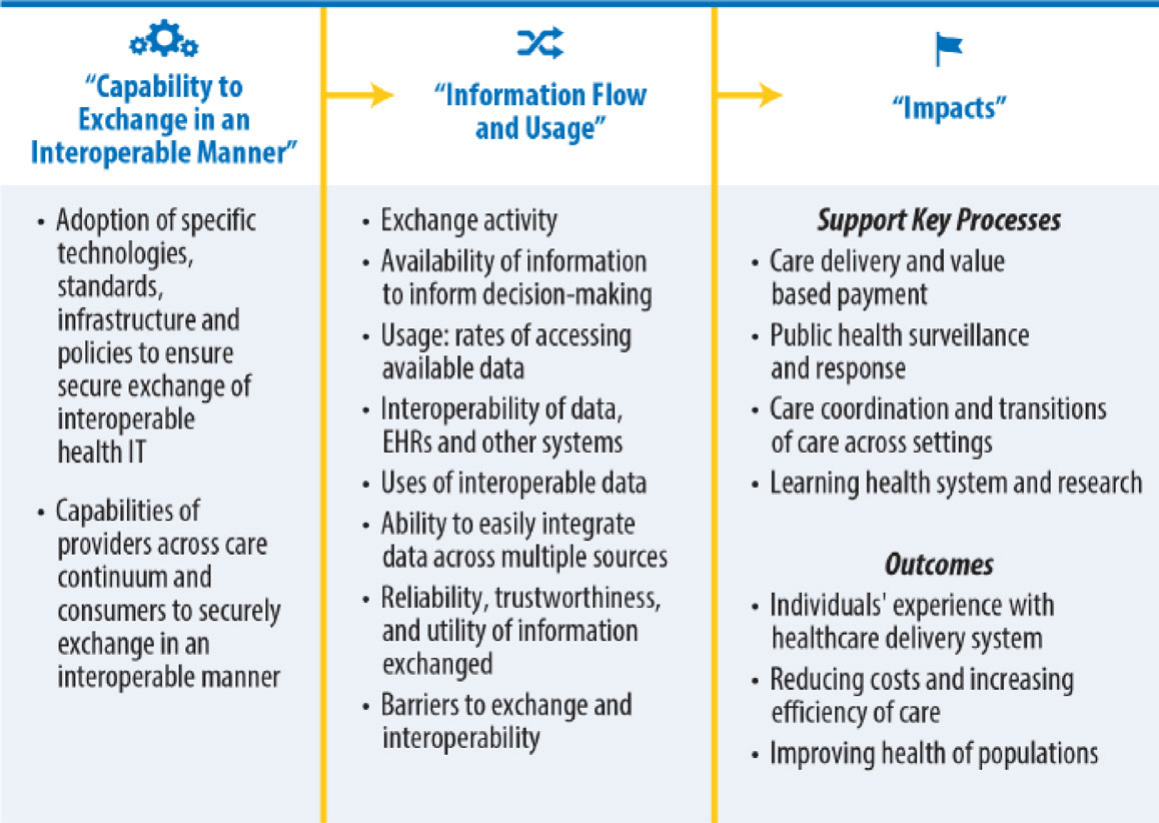
Measuring the impact and outcomes (NCHIT 2015; with permission).

The NCHIT also suggested a framework for measuring the effectiveness of the governance process for motivating and enabling the adoption of interoperable capabilities, by having identified the resulting impacts and outcomes. The framework is presented in Figure 11.

A forum established to identify collaborative requirements to enable effective interoperability in U.S. healthcare identified that the goal of governance should include increasing trust among potential exchange participants. This requires an understanding of what an organization needs to know about another organization in order to exchange data. A common understanding of the attributes of trust will minimize the need for one‐off trust agreements and contracts and permit the extension of existing trust communities to handle more use cases in the future. Once these attributes are discovered in relation to the exchange partner, and the means by which purported trust attribute values associated with that partner can be considered reliable, an organization should be expected to trust any organization that meets or exceeds their local policy requirements. This need is reflected in the 3 rules of the road principles summarized below.

#### Policy

Policy principles relate to the attitudes and concepts that determine the willingness of organizations to proactively share appropriate, accurate, and verifiable data, resulting in the creation of public good without compromising commercial considerations. Thus:

- No policy or business, or operational or technical barrier that is not required by law should be built to prevent information from appropriately flowing across geographic boundaries, IT solutions providers, or organizational boundaries in support of traceability.
- Where individuals/firms clearly instruct a data holder to release information about them to others, the data holder should comply with that directive.
- Participants should not compete on the availability of information. This principle makes sense in a healthcare‐related public service environment, but may not be transferrable to a commercial context such as the seafood sector.

Participants should not establish policies or practices in excess of law that limits the availability of data by another entity that is in compliance with applicable laws and these governance principles. Thus:

- Participants should grant firms, consistent with existing law, the ability to exercise choice over what data information these organizations collect from them and how the organizations use and share it.
- Participants should provide easily understandable and accessible information about organizations’ data practices, resulting in information transparency.
- Participants should secure and ensure responsible handling of other participants’ information.

#### Operations

Operations principles relate to data sharing policies and procedures among organizations. Thus:

- Participants should operate with transparency and openness, including making available information describing their electronic exchange capacity and services.
- Participants should promote inclusive participation and adequate stakeholder representation in the development of data policies and operations policies.
- There should be neutrality in the exchange of traceability data. That is, all exchange requests should be treated in the same open way and participants should not erect barriers to the authorized flow of information. For instance, an IT developer that has interoperable applications shall not prevent a user from using applications developed by competitors.

#### Standards

Participants should ensure that standards are prioritized, developed, and implemented to support the interests of the entire sector. Thus:

- Where available, vocabulary, content, transport, and security standards and associated implementation specifications are used.
- Standards should support data portability from one IT product to another.
- The development and implementation of technical requirements should enable the adaptation and incremental evolution of information exchange and technologies supporting exchange to meet current and future needs of users as standards evolve.
- Standards development and adoption should not unfairly provide an advantage to one firm/ organization over others.

### UK healthcare sector

The U.K. Government is committed to all patient and care records being digital, interoperable, and real‐time by 2020.

#### Governance

**Figure 12:**
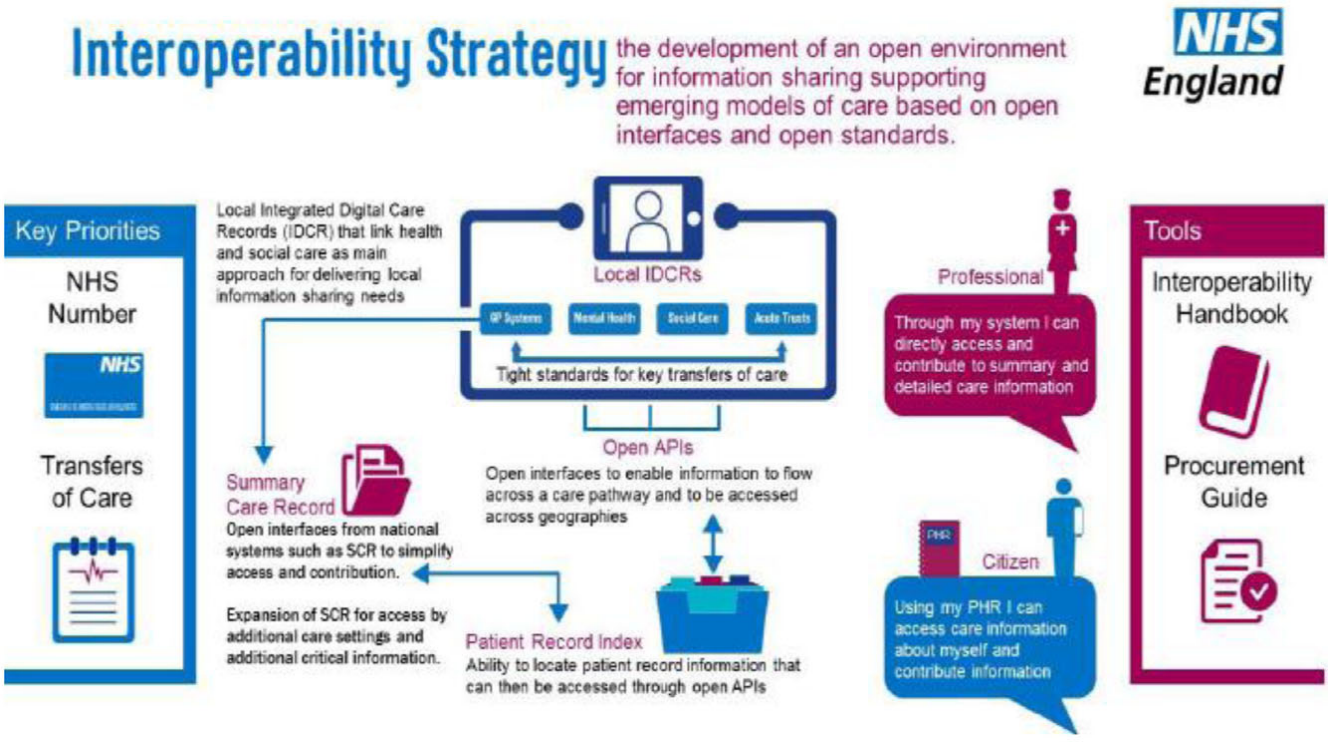
Schematic of UK health sector interoperability strategy (NHS England 2015. Contains public sector information licensed under the Open Government Licence v3.0. http://www.nationalarchives.gov.uk/doc/open-government-licence/version/3/).

To achieve this goal the U.K. government established the Natl. Information Board (NIB). Working closely with the Dept. of Health, the Natl. Health Service and NHS Digital, “the role of the Natl. Information Board is to put data and technology safely to work for patients, service users, citizens and the professionals who serve them. The NIB brings together national health and care organizations from the NHS, public health, clinical science, social care and local government, along with appointed independent representatives to develop the strategic priorities for data and technology” (NIB 2016). The governance process which NIB oversees revolves around working with regional health and care economies to meet the requirements set out in the Interoperability Strategy, shown in Figure 12.

#### Implementation

The government recommends that regional healthcare economies follow a 2‐phase process in developing and implementing an interoperability project plan. Government documents refer to the first step as “Where am I now?” and the second step as “Where am I going?”

- **Where am I now?**

First, each participant/partnership conducts a self‐assessment of its current state using the digital maturity index (NHS England 2016). The maturity index would be reviewed periodically to enable organizations to monitor their progress against the roadmap described below.

- **Where am I going?**

Secondly, each regional healthcare economy was challenged to prepare a roadmap specific to each participant/partnership by April 2016 which outlines the steps towards achieving interoperability. In the context of seafood this is equivalent to each supply chain developing its own roadmap to interoperability.

#### Guiding framework

It is also recommended that regional healthcare economies, each of which has a Commissioner who is responsible for its performance and management, use the questions summarized below to guide their development of a roadmap that suits their own particular situation. The questions fall into 5 overarching categories, each of which is listed below with examples.

- **Establish vision and scope**
  ○ Determine how interoperability will create value for the firm(s):
    ▪ Business drivers and technical issues which need solving
    ▪ What information needs to be shared? What are the priority use cases? Be specific regarding what functionality and what outcomes.
    ▪ What level of detail of information needs to be shared? Is it the same level with all supply chain members?
    ▪ Approach to information lifecycle management, for example what information needs to be retained and for how long?
  ○ Identify partners; discuss shared goals and vision alignment.
    ▪ Test vision with supply chain members, including shoppers/consumers. Consider setting up customer and consumer panels for the length of the project to ensure that it remains focused on creating value for these stakeholders.
    ▪ Review guidance/standards on interoperability (for example, KDEs) to inform the roadmap, and to avoid reinventing wheel.
    ▪ Understand cultural challenges:
      Ø Who will resist sharing data?
      Ø What internal processes and information flows will need to be adapted to facilitate interoperability, and is there agreement to this?
  ○ Prioritize which processes and information must be included, and which is nice to have. Make a clear link to objectives.
  ○ Prioritize order of delivery:
    ▪ What can be achieved quickly to mobilize commitment and build partnerships?
      Ø Focus where business benefit is greatest.
- **Review existing systems that will need to interoperate**
  ○ How interoperable are they currently? What changes are required, and at what cost?
  ○ Review:
    ▪ Whether existing proprietary systems include any restrictions on scope for interoperability, for example, licenses;
    ▪ What type of information are you trying to exchange, and
  ○ What are the dates for renewal of licenses and contracts with IT suppliers?
  ○ Does the solution need to be implemented rapidly or incrementally?
  ○ Does the solution need to suit a wide range of products, suppliers, and customers
  ○ Will the solution need to use architectural patterns that allow the systems to support information flows and provide updates to each other?
  ○ Test thinking against a range of suitably complex business problems to ensure that the proposed information solution(s) and architectural patterns are fit for purpose.
  ○ What skills can the firm/other partners deploy to support proposed patterns/solutions?
- **Establish vendor engagement strategy**
  ○ IT suppliers:
    ▪ Investigate which IT suppliers are already committed to interoperability
    ▪ Initiate links with IT supplier. Share plans and understand how well vendors align to the proposed development methodology/approach
    ▪ Then update high‐level business requirements and roadmap following feedback on market capability.
  ○ Establish governance structure and agree on funding and resources from services/partners, and undertake any required recruitment.
  ○ Ensure legal agreements are put in place to support any partnership and joint working arrangements for the program.
  ○ Agree on how to engage with stakeholders, including any outside direct members of the supply chain (NGOs/industry bodies, and so forth)
  ○ Agree on a procurement strategy and specification with partners, and select vendor.
- **Agree on information governance approach**
  ○ How will solution providers deliver choices, controls, and cybersecurity alongside legal compliance and access for regulators, including when operations occur in different international jurisdictions?
- **Revise and review**
  ○ Based on pilots and early adoption, consider refining or re‐defining manual and system processes and procedures.

## Summary of Findings

The topic of interoperability is attracting growing interest from businesses, industry organizations, including the seafood industry, and other stakeholders, including governments and NGOs. Intuitively and anecdotally, the benefits that are possible through implementation of effective and efficient interoperability can be significant. We analyzed 4 different industries to identify lessons learned about methods being used to implement interoperability at an industry level. Interoperability in the first industry examined—finance—essentially began in the 1970s with the establishment of SWIFT. The second industry—travel—began introducing interoperable solutions following the establishment of the OTA in 1999. The third industry—fresh produce—built on existing capabilities to establish interoperable traceability within 4 y of its launch in 2007 by using printed labels as the conduit to link businesses’ internal traceability systems. The fourth industry—healthcare—is in the process of implementing interoperability.

Arguably the most important and common finding across all industries is the need for a strong stakeholder‐driven governance system, along with a carefully crafted strategy that encourages the long‐term buy‐in of target stakeholders. This creates an enabling environment that is characterized by a series of common standards and protocols by which all involved stakeholders abide. That the developments which led to greatly improved traceability in all sectors other than healthcare were predominately industry‐funded shows that a clearly‐defined business case is also critical to success. Other important findings include the need for senior management support, because the process of implementing interoperability has to be driven from the top, and financial resources are required to establish ongoing activities. Senior management support is also required to address challenges that will inevitably arise during the implementation process. Other important findings include that while the most appropriate solution may differ by market, the enabling of interoperability relies on a series of common factors.

The development and implementation of interoperability solutions needs to take into consideration the market context and existing capabilities. It is essential that implementation of interoperability preserve the defining features of systems that enable effective and efficient traceability. The development of effective interoperability also stems from aligning strategic endeavours with key stakeholders, including standard setting organizations and regulatory agencies. Section “Conclusion,” which follows, elaborates on key enablers of interoperable traceability, which are achieved through the use of technologies that may differ by industry, market, and participant. The produce industry was able to achieve the required functions without mandating hardware and software requirements or standards, other than GS1 compliance, in part because products are not processed or transformed as they move along the supply chain. For example, whole trussed tomatoes are easily recognizable when harvested and shipped compared to when sold at the retail store.

Factors that may negate the potentially significant benefits that could otherwise be achieved by being part of a wider interoperable network include inappropriate implementation choices, or a premature regulatory mandate that forces the adoption of untimely or unsustainable solutions. The ineffective management of stakeholders that are more powerful than most and which may have entrenched opposing positions will negatively impact the development and implementation of industry‐level interoperability. As illustrated by the U.S. healthcare industry, powerful stakeholders with competing agendas can hamper the introduction of interoperable solutions. The need to address such situations underlines the importance of defining and targeting key stakeholders by establishing a pragmatic governance model, particularly in the early stages when quick wins are necessary to create the momentum required to engage stakeholders in an unproven initiative.

Of the 4 industries examined, finance, travel, and produce are most analogous to seafood. Each industry consists of widely varying operations in terms of scale, geographic location, capabilities, and ownership structures. The interoperability solution that suits each sector and business type is influenced by local infrastructure and participants’ technical capabilities. In produce, the extent to which transactions differ in size, perishability, and product transformation through packaging, and either aggregation or disaggregation as they move along the supply chain, is analogous to seafood.

Although compliance regulations are a factor that has driven the desire for increased interoperability, particularly in finance and produce, the impact of technology on enabling and driving the need for new services and solutions has been equally influential to the development of interoperable solutions. The rise of A2A transactions and GS1 standardization across industries are important cases in point. For example, because GS1 standards are equally applicable to multiple industries (for example produce, healthcare, and finance) and functions (for example traceability, quality control, and accounting) capabilities and lessons learned are readily transferrable. Thus, the seafood industry may be able to learn the most from the SWIFT‐managed ISO 20022 and adoption of GS1‐standardized communication protocols in enabling interoperability. The finance and produce models also foster the creation of industry‐specific interoperability standards, which were built on cross‐industry standards. Thus, the most valuable approach for enabling industries to benefit from interoperability is to use existing standards and protocols where possible.

Finally, a key takeaway from this study is that the effectiveness of initiatives established to guide the development and implementation of interoperability does not rely on them being enormous in scale or cost. For example, standardizing a traceable unit (the “case”) greatly simplified the implementation of interoperable traceability in the produce industry.

## Principles for Effective Interoperability

Determining the impact that technical and functional factors had on enabling interoperability in other industries, and identifying factors that will impact the design of a technology architecture suited to interoperable traceability in the seafood industry, allowed us to propose principles that must exist for effective and efficient computerized interoperability in the seafood industry. We summarize below these proposed principles, which are addressed in detail in *Interoperable Traceability Technology Architecture* produced and published by the GFTC (2017). The principles relate to factors that must exist for businesses to have the opportunity to create, and then capture value through the existence of syntactic and semantic interoperable traceability. Since traceability is just one function of ICT, the principles will also pertain to enabling effective, efficient interoperable ICT between computerized ICT systems per se. For a detailed review of factors impacting the design of a technology architecture suited to enabling interoperable traceability in seafood, refer to *Project to Develop an Interoperable Seafood Traceability Technology Architecture*: *Issues Brief* by Bhatt and others (2016).

Gupta (2008) describes a principle as a fundamental truth that provides a simple description of what is required to carry out functions and solve problems in systems that are themselves complex. Principles guide the development of systems that produce replicable outcomes, along with being scalable and sustainable, because they enable designers to understand the characteristics that a system must have to achieve a specific purpose. This section encompasses principles previously developed—*Software Engineering* by Ross and others (1975) and *Interoperability and Integration Fundamentals* by Reed (2006), for example—to ensure that computerized systems operate effectively and efficiently.

**Figure 13:**
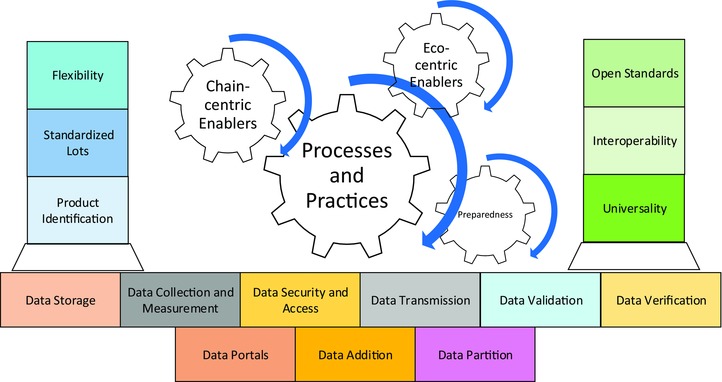
Interrelated principles for enabling interoperable seafood traceability.

The principles have been categorized into structural, operational, and integrative; the purpose being to guide practitioners through the complexities of designing an appropriate technology architecture. The principles describe requirements and solutions that are pivotal to enabling businesses to create and capture value from the existence of chain‐length interoperability. Adhering to the principles enables governments and advocacy groups to use traceability in the creation of public good. The following sections concisely describe each set of principles, with more detailed descriptions contained in the *Interoperable Traceability Technology Architecture* (Bhatt and Gooch 2017). A diagram illustrating how the 3 sets of principles together enable sustainable interoperability is shown in Figure 13.

### Structural principles

Structural principles—including open standards, standardized lots, and universality—are fundamental for establishing sustainable interoperability. Listed in Table 4, they must exist for networks of computerized systems to have the interoperable capabilities required to exchange syntactic and semantic data, then subsequently present that data in forms that allow the resulting information to be communicated and understood by multiple stakeholders.

**Table 4:** Structural principles for enabling interoperability (Bhatt and Gooch 2017)

Principle	Role in enabling interoperability
Interoperability	Syntactic and semantic interoperability required for the architecture to possess the extensibility capabilities required to ensure long term sustainability.
Universality	The ability must exist to seamlessly enable inputs and outputs to be exchanged between external and internal traceability systems that extend across supply chains.
Flexibility	So that it is not stifled by excessive rules and protocols, efficient interoperability relies on the ability to adapt to the needs of a diverse array of private and public stakeholders.
Open standards	The architecture's success and flexibility will rest on establishing a common ontology and industry standards to accurately define species, protocols, CTEs/KDEs, and so on.
Standardized lots	Defining a “standardized lot,” which may differ in size and format depending on the involved trading partners, is critical to enabling accurate and efficient communication.
Product identification	The architecture must enable unique global source‐, product‐, and location‐related identification codes to be attached to products as they move along supply chains.

### Operational principles

Operational principles—for example data collection and measurement, data validation, data verification, and data portals—describe the elements and listed in Table 5, which determine a system's functions and capabilities. Although critical to enabling businesses to create value from interoperability, their exact nature will be determined by users’ needs and available resources.

**Table 5:** Operational principles for enabling interoperability (Bhatt and Gooch 2017)

Principle	Role in enabling interoperability
Data addition	Data generated by each node in the supply chain, including previous lot numbers, must be linked.
Data portals	Access to portals for receiving, transmitting and retrieving data will depend on users’ role and relationship to the data.
Data partition	To minimize the need to silo data, permission to access data is conditional and controlled by a high level of security.
Data storage	Data is stored at the individual business level, with immediate visibility and accessibility enabled by secure portals.
Data transmission	Permissions are required to transmit data using software and hardware options that are chosen by individual users.
Data security and access	Security measures must be demonstrable and robust, with access to data determined by predefined arrangements.
Data collection and measurement	Data rigor ensured by use of defined KDEs and standardized measures to monitor performance, and validate authenticity.
Data validation	Key missing data are identified in the transmission process to ensure compliance with common standards, protocols, and so forth.

### Integrative principles

Integrative principles—including processes and practices, and preparedness—enable businesses and supply chains to use interoperability to create value. Although critical to enabling businesses to create value from interoperability and listed in Table 6, exactly how each of the integrative principles are implemented will be determined by users’ needs and available resources. Available resources include the availability and delivery of public institutions that shape the social, political, commercial, and physical environment within which individual businesses and supply chains operate.

**Table 6:** Integrative principles for enabling interoperability (Bhatt and Gooch 2017)

Principle	Role in enabling interoperability
Processes and practices	Processes defining the use of ICT are foundational and few in number. Practices for recording, storing, analyzing and distributing data differ across businesses and supply chains.
Eco‐centric enabling conditions	The cultural, technical, educational, and governance conditions that enable or constrain the implementation of solutions to interoperability differ markedly across regions of the globe.
Chain‐centric enabling conditions	The exact nature of the diverse and dynamic relationships that exist within and between businesses determine the solutions chosen to enable interoperable traceability.
Preparedness	The architecture must provide the ability for businesses and supply chains to accurately assess and select the options that best suit their requirements and produce the required ROI.

## Conclusion

Stemming from changing consumer demand, environmental concerns, government regulations, and other drivers, the seafood industry is increasingly competitive, global, and complex. The ability of businesses to proactively manage risks, reduce costs, and increase revenue relies on gaining transparency into activities that occur along the entire supply chain through the effective sharing of information. Verifying the accuracy and rigor of data exchanged within and between businesses in acquiring the necessary transparency for making informed management decisions requires effective interoperable information and traceability systems. Effective interoperability depends on the sharing of a common technology architecture, suited to enabling full chain interoperability, among the computerized systems used by businesses operating along the supply chain.

There is an emerging worldwide recognition of the need to develop computerized interoperable systems to achieve a series of potential benefits, including reduced costs and the ability to adapt faster to changing circumstances. A number of industries were examined and contrasted to investigate lessons learned that can guide seafood industry stakeholders through the process of determining how to establish an economically sustainable technology architecture that has the characteristics, functions, and capabilities required by the seafood industry for the purposes of traceability. Lessons learned from this investigation were then distilled into a series of principles that can guide the development of computerized interoperability in any industry, not only seafood. The key learning from this study is that the principles must be followed to enable the development of effective computerized interoperability systems. The way in which the principles are actualized for the required functions and capabilities at an industry and enterprise level, however, may differ depending on environmental and technical considerations.

To illustrate the relationships that exist between each principle and industry‐ versus enterprise‐level consideration, we categorized the principles as structural, operational, and integrative. Structural principles address how interoperability is actualized at the industry level, for example through development of common standards and protocols that are incorporated in the development of software, which must exist and are fundamental requirements in the development of interoperability solutions. In all of the sectors, structural principles are enabled and maintained through a defined governance process. The finance industry's governance process, for example, is designed to ensure that ISO 20022 standards are adhered to during the development and implementation of interoperable solutions. The governance process also ensures that regardless of who or where solutions are enacted worldwide, they integrate succinctly with current systems and capabilities to address an identified need.

The way in which operational and integrative principles are enabled and actualized will depend upon the processes and capabilities of individual businesses as well as their access to technology. The PTI, for example, provides businesses that have limited access to computerized technologies or broadband internet an ability to exchange data and information through printed labels to be carried on traded products. Businesses whose computerized systems connect directly with those of their trading partners can use practices such as advance shipment notices to capture greater value from interoperable traceability. Similar levels of adoption and potential value were identified in the other sectors analysed. NGOs that are expert in implementing innovative traceability and technology solutions may have an important role to play in actualizing interoperable seafood traceability in those emerging economies where access to modern technology infrastructure and capabilities is severely limited.

## Authors’ Contributions

The lead authors were Martin Gooch and Tejas Bhatt. Benjamin Dent contributed to the analysis of interoperability in the health, travel, and financial industries. Gil Sylvia contributed to the analysis of the PTI and development of the principles of interoperability.
